# Establishment of Epithelial Attachment on Titanium Surface Coated with Platelet Activating Peptide

**DOI:** 10.1371/journal.pone.0164693

**Published:** 2016-10-14

**Authors:** Shiho Sugawara, Masahiko Maeno, Cliff Lee, Shigemi Nagai, David M. Kim, John Da Silva, Masazumi Nagai, Hisatomo Kondo

**Affiliations:** 1 Department of Restorative Dentistry and Biomaterials Sciences, Harvard School of Dental Medicine, Boston, Massachusetts, United States of America; 2 Department of Oral Medicine, Infection and Immunity, Harvard School of Dental Medicine, Boston, Massachusetts, United States of America; 3 Department of Prosthodontics and Oral Implantology, Iwate Medical University, School of Dental Medicine, Morioka, Iwate, Japan; 4 Department of Adhesive Dentistry, The Nippon Dental University, Chiyoda-ku, Tokyo, Japan; Virginia Commonwealth University, UNITED STATES

## Abstract

The aim of this study was to produce epithelial attachment on a typical implant abutment surface of smooth titanium. A challenging complication that hinders the success of dental implants is peri-implantitis. A common cause of peri-implantitis may results from the lack of epithelial sealing at the peri-implant collar. Histologically, epithelial sealing is recognized as the attachment of the basement membrane (BM). BM-attachment is promoted by activated platelet aggregates at surgical wound sites. On the other hand, platelets did not aggregate on smooth titanium, the surface typical of the implant abutment. We then hypothesized that epithelial BM-attachment was produced when titanium surface was modified to allow platelet aggregation. Titanium surfaces were coated with a protease activated receptor 4-activating peptide (PAR4-AP). PAR4-AP coating yielded rapid aggregation of platelets on the titanium surface. Platelet aggregates released robust amount of epithelial chemoattractants (IGF-I, TGF-β) and growth factors (EGF, VEGF) on the titanium surface. Human gingival epithelial cells, when they were co-cultured on the platelet aggregates, successfully attached to the PAR4-AP coated titanium surface with spread laminin5 positive BM and consecutive staining of the epithelial tight junction component ZO1, indicating the formation of complete epithelial sheet. These in-vitro results indicate the establishment of epithelial BM-attachment to the titanium surface.

## Introduction

The outcomes of dental implants can be classified into three distinct categories: failure, survival and success. Implants are regarded as a *failure* if it is removed for any reason, with clinical mobility being an absolute indication for their removal. *Survival* indicates that the implants are present in the oral cavity and functioning [1], with the survival rate reported to be around 95.5–97.8%. Implants in the survival category achieve *success* if they meet specific criteria, including a lack of biologic and/or technical complications [1], achieving an adequate amount of keratinized mucosa and soft tissue coverage, and patient satisfaction [2]. According to these reports, the *success* rate of implant treatment is estimated to be around 81.3–93.8%. As implant *survival* has remained very high, the implant *success* has become increasingly adopted as the standard in assessing clinical outcomes.

A challenging complication that hinders the success of implant treatment is peri-implantitis. A common cause of peri-implant mucositis and peri-implantitis may be the lack of tight epithelial seal around the implant and abutment junction [3–5]. This lack of epithelial sealing allows for the entry and proliferation of bacteria, leading to soft tissue inflammation and alveolar bone resorption in a process similar to periodontal disease when the epithelial seal in natural teeth is disrupted. Peri-implantitis significantly affects the prognosis of implants when it is accompanied by alveolar bone loss [6]. The overall frequency of peri-implantitis has been reported to be 28%-56% of subjects and 12–43% of implant sites [7]. To improve the success rate of implant treatment, a strong epithelial seal should prevent potential bacterial invasion and growth.

The periodontium of natural teeth is protected from the bacterial invasion by the firm sealing of the junctional epithelium (JE) which attaches to tooth surface via a basement membrane (BM) [8]. However, the peri-implant epithelium (PIE) is clinically recognized to have poor attachment with few BM attachments [9]. Ultrastructural study showed that the baso-apical orientation was such that the BM faces the connective tissue side and apical cell membrane faces the implant side [10]. As Larjava et al. discussed in their review, [11] the PIE appeared to just “lean” on the implant surface with the apical side of epithelial cells without a strong attachment. Although the other studies have shown the BM attachment of PIE to implant surface with topological [12] or chemical modification [13, 14], the attached area was restricted to the bottom third. This does not include the coronal gingival margin where epithelial attachment is desired. Thus, the direct attachment of PIE to the titanium implant or abutment surface via a BM is likely the key to prevent peri-implantitis and achieve dental implant success.

When epithelial attachment is lost due to periodontitis around teeth, gingival epithelium can be restored with functional BM-attachment to dental root surfaces when the root surface is scaled to expose fresh cementum, on which platelets can form activated aggregates [15, 16]. Platelets provide the comprehensive machinery for a fibrin clot in epithelial healing with the proper apico-basal polarization. In normal physiologic wound healing, epithelial cells migrate from the wound edge beneath platelet-fibrin clot to restore continuity of epithelial sheet with its BM by attaching to the opposite side of the clot. Platelet aggregation is the first step leading to fibrin clotting and granulation. Thus, if a modified titanium surface activates platelet aggregation, fibrin clot and granulation tissue may follow and the epithelial BM-attachment can be promoted on the titanium surface.

Although several studies have demonstrated actual attachment of PIE at the coronal area, they have limitations for clinical application. Werner et al. showed PIE attachment via the BM at coronal gingival margin area of titanium surface, but it was limited to only a porous titanium surface [17]. Studies have shown that porous implant collar surfaces allow for significantly higher bacterial attachment compared to smooth-polished surfaces [17–19]. Atsuta et al. [20] also indicated extended PIE attachment to the coronal area in mice by topical application of IGF-I. However, it required repeated applications of IGF-I over a two-week period which is unrealistic in clinical use. As such, we designed a chemical coating on smooth titanium surface that could induce actual attachment of PIE in the clinical loading protocol of dental implants and abutments.

In this *in vitro* study, we employed a protease activated receptor 4-activating peptide (PAR4-AP), to promote platelet-fibrin clot aggregation on the titanium surface. PAR4-AP mediates the thrombin-activated platelet fibrin clot formation. Thrombin is a serine-protease that cleaves the N-terminus of the protease activated receptor (PAR), which in turn acts as a tethered ligand to activate PAR on the platelet surface for the clot formation. The PAR4-AP sequence of AYPGKF was selected because it has comparable activity to thrombin against PAR4 [21, 22]. In this study, we hypothesized that epithelial BM-attachment was produced when titanium surface was modified to allow platelet aggregation. The aims of this study are to demonstrate: i) rapid formation of platelet aggregation on the PAR4-AP coated titanium surface; ii) release of epithelial chemoattractants and growth factors from the aggregates on the titanium surface; and iii) ultrastructural and immunocytochemical proofs of epithelial BM attachment to the PAR4-AP coated titanium surface.

## Materials and Methods

Harvard University Faculty of Medicine Office of Human Research Administration determined that this study was not a human subject research as defined by DHHS and FDA regulations (Protocol number: RNI16-0186).

### Immobilization of PA linker on titanium surface

For imaging studies, titanium foil (GalliumSource, LLC, CA, USA) was cut into 1.0 cm x 1.0 cm x 0.05 mm squares. Round discs (5 mm in diameter) were prepared for quantitative determination of platelet aggregation and epithelial cell attachment in 96-well plate format. The titanium pieces were ultrasonically cleaned in 0.5% sodium dodecyl sulfate (SDS; Sigma, MO, USA), deionized water, acetone (Sigma, MO, USA) and ethanol (Sigma, MO, USA), sequentially, for 20 minutes in each solvent. The cleaned titanium pieces were incubated for 3 hours in 1mM PA linker (10-CDPA with carboxyl end, Dojindo Molecular Technology, MD, USA) solution in ethanol with gentle rocking at 70°C. The titanium pieces were washed in ethanol 4 times, air-dried, and heated at 120°C for 24 hours in a drying oven.

### Coupling with PA linker and PAR4-AP

The carboxyl residue of the linker was activated by rotating the titanium pieces at room temperature for 2 hours in 0.2M N-hydroxysuccinimide (NHS; Sigma, MO, USA) and 0.25M 1-ethyl-3-(3-dimethylaminopropyl) carbodiimide (EDC; Simga, MO, USA) dissolved in dimethylformamide (DMF; Sigma, MO, USA). After washing 4 times in DMF, the linker was coupled with PAR4-AP (AYPGKF-NH_2_; Peptides International, KY, USA) by rotating the titanium pieces at room temperature for 70 minutes in DMF containing 0.1mM PAR4-AP. Afterwards, the titanium pieces were washed 4 times with DMF, then air-dried and stored at 4°C.

### Preparation of PRP

Citrate-anticoagulated whole blood was purchased from BioreclamationIVT (NY, USA). Each whole blood was collected from a single donor without any identification involved. Platelet-rich plasma (PRP) was obtained by centrifugation at 180 g for 15 minutes at room temperature. A part of PRP was recentrifuged at 1500 g for 10 minutes to obtain platelet poor plasma (PPP). Platelet count was done on a hemocytometer.

### Experimental culture format and assay flow

**Fig 1:**
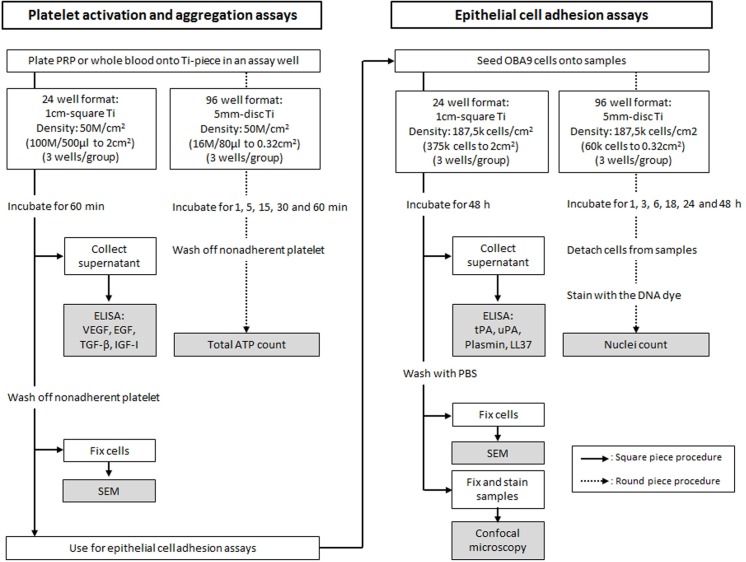
Schematic experimental flow of platelet activation and aggregation, and epithelial cell adhesion assays. Schematic of experimental flow of platelet activation and aggregation, and epithelial cell adhesion assays.

Quantitative evaluation of platelet and whole blood aggregation, and the following epithelial attachment was done on 5mm titanium discs. Histological evaluation of PRP and whole blood aggregation and epithelial attachment was done on 1cm titanium squares. Experimental culture formats and flow are summarized in Fig 1.

### Kinetic adhesion of PRP and whole blood to titanium surface

Culture condition of kinetic adhesion of PRP and whole blood is shown in Fig 1. PRP and whole blood were inoculated onto the titanium discs with and without surface coating (n = 3 for each group) and incubated at 37°C for 1, 5, 15, 30 and 60 minutes. At the end of incubation, unbound cells were washed off with PBS. The titanium pieces were then transferred to a new assay well of 96-well white opaque plate, and ATP content of the adhered cells was measured by using a kit (CellTiter-Glo Luminescence Cell Viability Assay, WI, USA).

### Ultrastructure of PRP and whole blood aggregation

PRP and whole blood were inoculated onto 1cm titanium squares in the 24-well format (Fig 1). At 60 minutes of incubation, unbound platelets and blood cells were washed off with PBS, and the cells adhered on the titanium surface were fixed in 4% paraformaldehyde (PFA), washed in water, and dehydrated in ethanol series to 100% (75%, 80%, 85%, 90%, 95% and 100%, 20 min in each incubation). Ultrastructure of the cells on the titanium surface was imaged by scanning electron microscopy (SEM; Zeiss Supra 55VP field emission scanning electron microscope; ZEISS, Oberkochen, Germany).

### Cytokine release from PRP

Culture supernatant was collected from 60 minutes incubation of PRP culture in the 24-well format (Fig 1). EGF, IGF-I, TGF-β and VEGF in the supernatant was measured by using corresponding ELISA kits (R&D Systems, MN, USA). Triplicate aliquots of each culture supernatant were quantitated in each ELISA.

### Human gingival epithelial cells

Usage of human gingival epithelial cells (OBA9) was permitted by Dr. Murakami at Osaka University School of Dentistry. Cells were provided by Dr. Kawai at the Forsyth Institute (Cambidge, MA, USA). The cells were maintained in keratinocyte-serum free medium (SFM; Thermo Fischer Scientific, MA, USA) at 37°C in 5% CO_2_ and 95% atmospheric air. The original media-supplements of EGF and bovine pituitary extract, and antibiotics were not included in the present study.

### Kinetic epithelial adhesion to titanium surfaces

Kinetic adhesion of epithelial OBA9 cells was done in the 96-well format (Fig 1). OBA9 cells were co-cultured on the titanium discs with and without surface coating (n = 3 for each group) on which PRP was previously incubated for 60 minutes. Unbound platelets were washed off with PBS from the titanium discs before OBA9 cells were inoculated. OBA9 cells were then co-cultured on the titanium disc for 1, 3, 6, 18, 24 and 48 hours. At the end of each time point, unbound cells were washed off with PBS. Cells remained on the disc were transferred to a new 96-well plate with V bottom. Cells were detached from the titanium disc by enzymatic digestion in 50μl of trypsin-EDTA (TrypLe select, Thermo Fisher Scientific, MA, USA) for 5 minutes. Titanium discs were removed after rinsing with 50μl of fresh tryspin-EDTA. Then, 100μl of DNA-dye solution containing 2.5μg/ml of Hoechst 33342 (Thermo Fisher Scientific, MA, USA) in 4% PFA-0.2% Triton X100 was added to the cell suspension. After staining with the DNA dye for 10 minutes, cells were centrifuged to remove unbound DNA-dye, washed and resuspended in 100μl of PBS, transferred to a new assay well of 96-well black opaque plate, and DNA-fluorescence was measured at 360nm/excitation and 430nm/emission on a plate reader (FilterMax F5; Molecular Devices, CA, USA).

### Ultrastructure and immunocytochemistry of OBA9 epithelial cells on titanium surfaces

Histological observation of OBA9 epithelial cells was done in the 24-well format (Fig 1). Likewise in the 96-well format, OBA9 cells were inoculated on 1cm titanium squares from PRP aggregation assay, incubated for 48 hours, and processed for SEM. A part of the OBA9 specimen was not dehydrated in the ethanol series to avoid nonspecific staining in immunocytochemistry. BM and TJ proteins of Ln5 and ZO1, respectively, were stained with fluorescence-labeled antibodies (laminin-5 antibody, DyLight 488 conjugate; ZO1 antibody, Alexa Fluor 594 conjugate; Thermo Fisher Scientific, MA, USA). The cell nuclei were counter-stained with DAPI (FluoroshieldTM with DAPI; Sigma, MO, USA), and imaged under confocal microscopy (Zeiss LSM 710 confocal microscope; ZEISS, Oberkochen, Germany). Size of immunostaining positive area was determined by using Java image processing program ImageJ.

### Secretions of fibrinolytic and antibacterial substances from OBA9 cells

Culture supernatant of the co-culture of OBA9 cells and PRP pre-incubated titanium in the 24-well format was collected for ELISA quantitation for tPA (R&D Systems, MN, USA), uPA (IBL, MN, USA), plasmin (Creative Diagnostics, NY, USA) and LL37 [23]. Triplicate aliquots of each culture supernatant were quantitated in each ELISA.

### Statistical analysis

All quantitative measurements were done in triplicates, and each data point represents mean ± SD (standard deviation). Statistical significance between control and experimental group was determined by unpaired, 2-tailed t-test with unequal variance. Also, the data including more than two groups were analyzed with one-way ANOVA and post hoc q-test. Significance levels were set to p < 0.1 and p < 0.05.

## Results

### Platelets and whole blood aggregation on titanium surface

**Fig 2:**
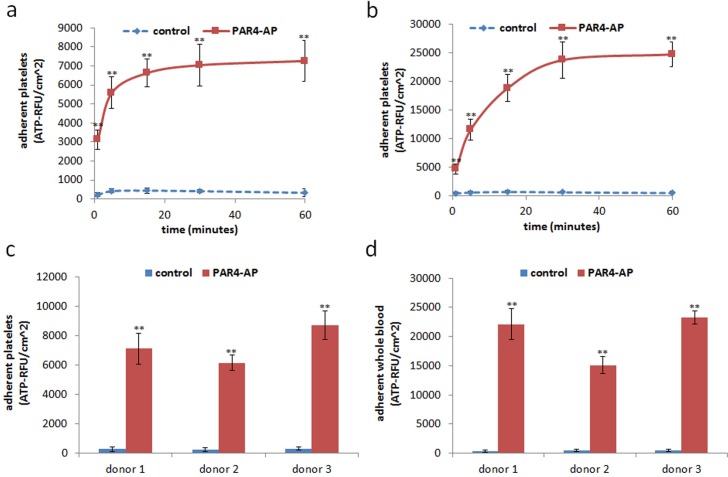
Quantitation of PRP and whole blood aggregation on the titanium surfaces. PRP and whole blood aggregation was quantified by total cellular ATP in a time course at 1, 5, 15, 30 and 60 minutes (a,b) and at a plateaued time point of 60 min (c,d). Values are mean ± SD of three independent cultures of PRP and whole blood from one representative donor (a,b) and three independent donors (c,d). ** indicates a significant difference with control at the corresponding time point (**, *p < 0*.*01*). Supplemental data is published in http://dx.doi.org/10.7910/DVN/FDMPZY.

**Fig 3:**
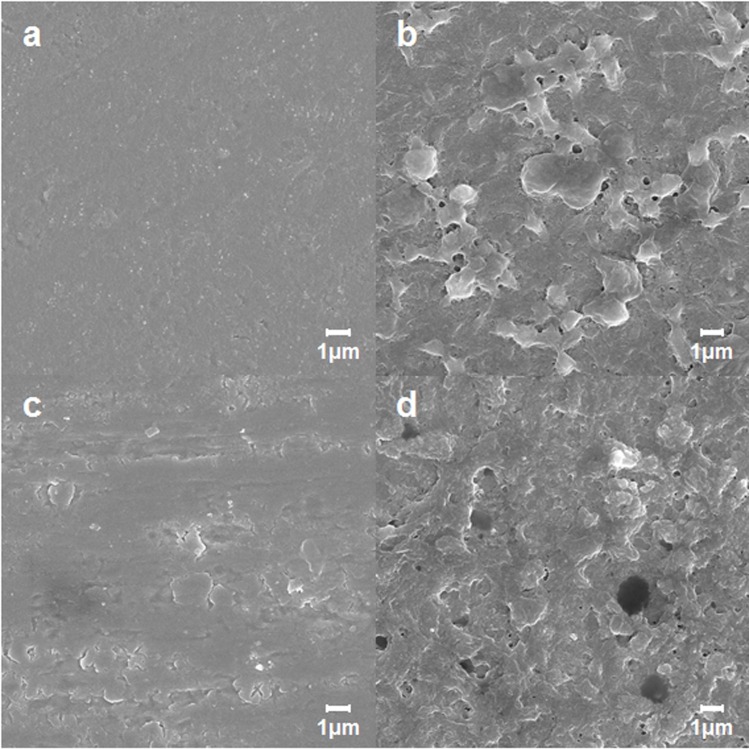
SEM ultrastructure of PRP and whole blood aggregation on the titanium surfaces. PRP (a,b) and whole blood (c,d) were incubated for 60 minutes on the titanium surfaces with untreated control (a, c) and PAR4-AP coating (b,d). SEM micrographs were taken at the magnification of 10,000x. Supplemental data is published in http://dx.doi.org/10.7910/DVN/FDMPZY.

A smooth titanium surface allowed little adhesion of cells in either PRP or whole blood at every time point (Fig 2A and 2B). On the other hand, the PAR4-AP coated titanium surface allowed rapid increase in cell adhesion from both PRP and whole blood. The accumulation plateaued in 30 minutes. The effect of PAR4-AP was not donor dependent; comparable accumulation was observed in the all specimens collected from three independent donors (Fig 2C and 2D). SEM captured the dense aggregation of platelets on the PAR4-AP coated titanium surface in both PRP and whole blood (Fig 3C and 3D, respectively). PA-linker alone was inactive on the cell adhesion and aggregation (shown in supplemented figure). PAR4-AP induced platelet aggregation was accompanied by robust increases in the secretions of epithelial chemoattractants (IGF-I, TGF-β) and growth factors (EGF, VEGF) that were rapidly released in 60 minutes incubation (Table 1). Baseline levels of cytokines in the PPP of the corresponding PRP were comparable or lower than supernatant of PRP in the control culture.

**Table 1:** Cytokines in the supernatant of PRP cultured on titanium surfaces.

analyte	Control	PAR4-AP	PPP
EGF	90 ± 4^a^	331 ± 16^b^	66 ± 4^a^
IGF-I	98 ± 6^a^	262 ± 28^b^	83 ± 15^a^
TGF-β	33 ± 1^a^	203 ± 10^b^	14 ± 6^a^
VEGF	18 ± 4^a^	299 ± 51^b^	18 ± 7^a^

Concentration unit is pg/ml. Value represents mean ± SD of three independent cultures of aliquots of PRP from one representative donor. The same superscripts indicate no significant difference (p < 0.01). Supplemental data is published in http://dx.doi.org/10.7910/DVN/FDMPZY.

### Epithelial attachment to titanium surface

**Fig 4:**
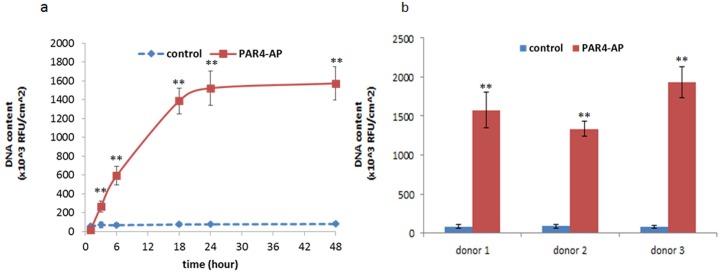
Adhesion of OBA9 epithelial cells to PRP pre-incubated titanium surfaces. Adhered OBA9 cells were counted by nuclear DNA staining with Hoechst 33342. Each data point represents mean ± SD of three independent cultures. Kinetic adhesion was determined in the titanium that was pre-incubated with PRP from one representative donor (a). The adhesion assay was triplicated at 60 minutes with PRP from different donors (b). ** indicates a significant difference than control at the corresponding time point and in the corresponding donors (**p < 0.*01*). Supplemental data is published in http://dx.doi.org/10.7910/DVN/FDMPZY.

Adhesion of OBA9 epithelial cells was examined on the titanium discs that were pre-incubated with PRP (Fig 1). PRP was passed through 4μm-pore mesh to minimize the contamination of blood cells having nuclei. On the control titanium surfaces where platelets did not aggregate (Fig 3A), nuclei count of OBA9 cells was low at a marginal baseline level during the time course (Fig 4A). On the PAR4-AP coated titanium surface, where PRP densely aggregated (Fig 2C), time-dependent increase in the adhesion of OBA9 epithelial cells was significant (Fig 4A). The nuclei count of OBA9 cells plateaued by 48 hours on the PAR4-AP coated surface. Donor-independent effect of the PAR4-AP coating on the induction of the epithelial cell adhesion was confirmed at 48 hours (Fig 4B).

**Fig 5:**
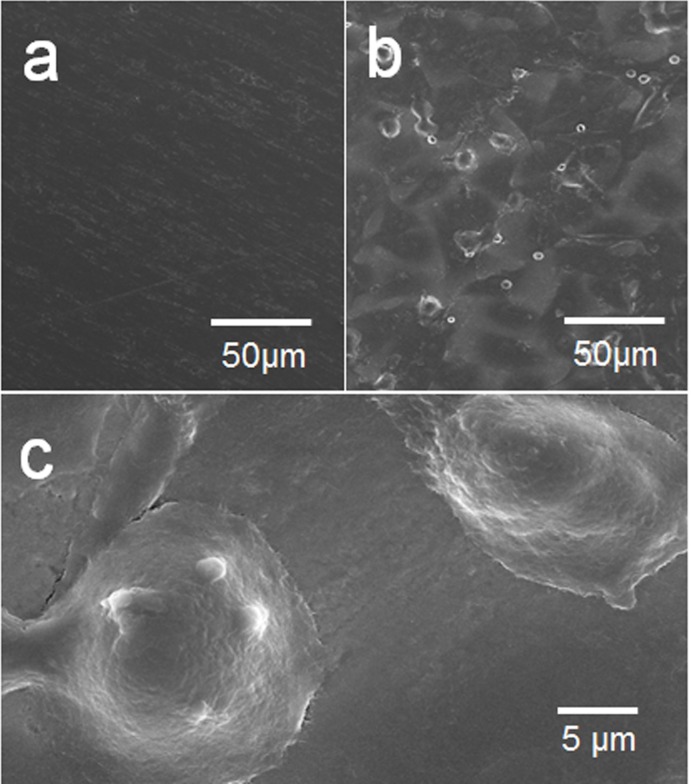
SEM ultrastructure of OBA9 epithelial cells on the PRP pre-incubated titanium surfaces. Representative SEM micrographs of OBA9 epithelial cells on the untreated control (a) and PAR4-AP coated titanium (b,c) surfaces at the magnifications 500x (a,b) and 5000x (c). Supplemental data is published in http://dx.doi.org/10.7910/DVN/FDMPZY.

**Fig 6:**
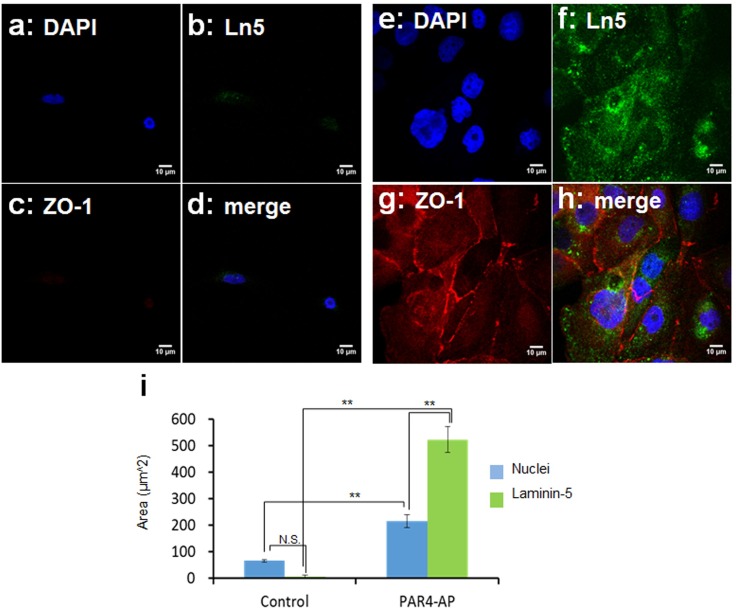
Immunocytochemical staining of basement membrane-laminin5 and tight junction-ZO1. OBA9 epithelial cells were co-cultured on PRP pre-incubated titanium surfaces (untreated: a-d; PAR4-AP coated: e-h) for 48 hours. Representative confocal micrographs of laminin-5 (Ln5, green) and ZO1 (red). Nuclei were counter-stained with DAPI (blue). Three cells were randomly selected from independent micrographs (n = 3) for the quantification (i). Statistical p-value between untreated-control and PAR4-AP coated titanium in a one-way ANOVA and post hoc q-test (**p < 0.01). Supplemental data is published in http://dx.doi.org/10.7910/DVN/FDMPZY.

The mode of epithelial attachment was identified by SEM ultrastructure and immunocytochemistry. In accordance with the low nuclei count, SEM captured few cells on the untreated control surface (Fig 5A). These cells failed to show laminin-5 positive BM (Fig 6B and 6D). It was confirmed that little cells existed on a smooth titanium surface with no BM attachment. On the other hand dense colonies with flat epithelial cell-like morphology were seen on the PAR4-AP coated surface (Fig 5B). These cells showed flattened large nuclei with spreading cytoplasm. A replication front of epithelial cells shows the morphology of expanding BM (Fig 5C). Epithelial BM-attachment on the PAR4-AP surface was identified with BM-Ln5 (Fig 6). Widely spread green-Ln5 area was shown on the PAR4-AP surface (Fig 6B and 6F). Fig 6I quantitated the significant increase in the laminin-5 positive area on the PAR4-AP surface. These laminin-5 positive cells also show consecutive staining of ZO1 (Fig 6C and 6G), a marker protein of epithelial tight junction, indicating a complete epithelial sheet.

### Fibrinolytic and antimicrobial molecules in epithelial conditioned medium

Dense platelet aggregates were not seen when the OBA9 cells occupied the PAR4-AP surface suggesting an aggregate dispersing activity in the epithelial conditioned medium. Fibrinolytic tPA, uPA and the resultant plasmin were measured in the conditioned medium.

Significant increases in tPA, uPA, plasmin and LL37 were induced in the co-culture of OBA9 cells and PRP aggregated PAR4-AP surface (Table 2).

**Table 2:** Pro-fibrinolysis mediators and antimicrobial peptide in epithelial cell supernatant.

analyte	Control	PAR4-AP	
tPA	734±85	3258±261**	pg/ml
uPA	227±85	2593±307**	
plasmin	32±12	285±36**	ng/ml
LL37	77±13	303±52**	

Concentration unit is pg of analyte per μg total protein of PRP and OBA9 cells. Values are mean ± SD of three independent cultures of PRP from one representative donor.

** indicate a significant difference with control at the corresponding time point (**p < 0.01). Supplemental data is published in http://dx.doi.org/10.7910/DVN/FDMPZY.

## Discussion

Osseointegation of a titanium implant is typically enhanced with micro-surface roughness [24]. On the other hand, the implant and abutment collar surfaces are kept polished and smooth, primarily to reduce active bacterial colonization. Although this passive antifouling eventually allows bacterial invasion and growth, there are no other active antifouling mechanisms that block bacterial invasion. Natural teeth have an epithelial seal provided by the JE attachment to the tooth surface which prevents bacterial invasion to the periodontium [25]. If a firm epithelial attachment is reproduced at the implant or abutment surface prior to bacterial invasion, the peri-implant epithelium can be the ideal antifouling defense.

The present study showed that a smooth titanium surface did not allow for the attachment of platelets (Fig 3A and 3C). This was not surprising because a nonthrombotic surface is a critical requirement for medical implants, such as vascular stents, heart valves, and ventricular assist devices. However, platelet attachment is the critical step for platelet aggregation and the following fibrin clot that promote epithelial wound healing. Thus, the failure of platelet attachment and aggregation can explain the failure of epithelial attachment to a titanium implant surface.

We utilized platelet-fibrin clot for surgery wound healing at dental implantation site *in-situ*. The implant and the abutment collar surfaces locate to the vicinity of oral cavity where air is available for fibrin coagulation via thromboplastin-extrinsic pathway. Therefore, the intrinsic coagulation pathway via PAR4-AP activated platelet aggregation could be enhanced by the extrinsic pathway at the implantation site *in-situ*.

As we expected epithelial cells did attach on the platelet aggregated titanium surface via BM. The attachment process of OBA9 cells to the PAR4-AP coated surface included transmigration through dense platelet aggregate layer (Fig 3B) and adhesion of the BM-Laminin5 to the titanium surface (Fig 6F). Initiation of migration towards platelet aggregated titanium surface could be explained by the burst of chemokine release including EGF and IGF-1 (Table 1); Epithelial cells migrate towards IGF-I coated culture surface with the induction of laminin5 expression [26]. Another phenomenon that we should discuss here is the disappearance of dense platelet aggregates from the titanium surface when OBA9 cells occupied. This is explained by tPA, uPA and the resultant plasmin [27] that were released from OBA9 cells (Table 2). Plasma plasminogen could deposit on the platelet aggregates and become activated to plasmin by tPA and uPA that were released from the OBA9 cells.

A practical feasibility of the present approach on the platelet induction *in-situ* would be the availability of fully effective PAR4-AP peptide. *In-situ* application of the peptide for platelet activation can overcome the significant limitation, typical of *in-vivo* applications of protein and peptide ligands; their biological half-lives are often too short to fully utilize their potential activity. However, PAR4-AP first meets its target of blood platelets in an implant surgery site. Therefore the *in-vivo* half-life would not be a limitation for PAR4-AP. In addition to the removing the limitation of the biological half-life/*in vivo* stability issue, the use of PAR4-AP peptide has many additional benefits, including safety, biocompatibility, cost, and ease of handling. The peptides are chemically synthesizable, xeno-free, non-antigenic, and resistant to heat and chemical sterilization. Chemical synthesis of PAR4-AP allows for a cost effective method of production compared to recombinant protein synthesis. These properties give PAR4-AP significant potential in clinical applications.

The OBA9 cells used in the present study were SV40-immortalized cell line established from human inner gingival epithelium adjacent to the tooth surface [28]. OBA9 cells retain epithelial like morphology as well as the expression of epithelial marker proteins, [29] such as laminin-5 and ZO1 which we were able to demonstrate. The significance of laminin-5 cannot be understated for BM epithelial adhesion and function. The consecutive staining with ZO1 in the epithelial sheet on the PAR4-AP coated titanium surface also supports the function of PAR4-AP induced epithelial attachment as a physical barrier for pathogens.

We would expect the treatment of the implant surface to improve the outcomes of implant treatment. The impact of cell adhesion to the surface of the implant would be the improvement in the ability of the implant site to resist bacterial infiltration which would result in a decrease in the inflammatory response around the implant. The reduction in the inflammatory response will result in a reduction of bone loss around an implant overtime and a reduction in the incidence of peri-implantitis. This will result in an improvement of the life span of an implant. Companies are in a continuous improvement phase for their implant products. They are looking for solutions to problem like peri-implantitis which currently has no reliable treatment approach.

There are some limitations in this *in-vitro* model. The first is that the epithelial cells were dispersed in free-single cell suspension, which only occurs in *in-vitro* conditions. Normal epithelial cells exist in an epithelial sheet where cells are tightly attached to one another with desmosomes [30]. Epithelial cells at a wound edge *in-vivo* need to be liberated of the desmosomal linkage to migrate out from the epithelial sheets. Although the single-cell suspension has been widely accepted in various *in-vitro* studies, each epithelial cell can freely migrate without desmosomal restraint. Another limitation may be the passive sedimentation of epithelial cells towards titanium surface due to gravity. Epithelial cells were applied onto titanium pieces with the exposed clot surface facing upward in the bottom of culture well. The next step will be to fully replicate an active epithelial migration towards the platelet-clotted titanium surface against the forces of gravity. However, these limitations do not suggest that active epithelial cell migration did not occur as well. The OBA9 cells migrated through the clot layers to reach the modified titanium surface. Increased levels of epithelial chemoattractants also suggest active migration of OBA9 cells. Another limitation is that fibrin coagulation could not be evaluated due to the presence of the anticoagulant citrate in *in vitro* PRP and whole blood. In addition, the process of fibrin cot formation is complex with many factors and steps that may have unpredictable interactions *in vivo* that cannot be accounted for *in vivo*.

In conclusion, the results of this *in-vitro* study successfully demonstrated that epithelial-BM attachment is induced when platelets were activated to form aggregation on the modified surface. Based on the versatility of PAR4-AP for chemical modification and resistance under physical insults such as heat or surface modifications, the present surface treatment may provide a functional material that can achieve a more successful dental implant treatment.
